# Maxillary neoplasms in four dromedary camels

**DOI:** 10.3389/fvets.2023.1153398

**Published:** 2023-06-28

**Authors:** Abdelazim Ibrahim, Mohamed Zabady, Ayman El Nahas, Ahmed Aljazzar, Fahad Al-Hizab, Mahmoud Kandeel, Brian F. Porter

**Affiliations:** ^1^Department of Pathology, College of Veterinary Medicine, King Faisal University, Al-Ahsa, Saudi Arabia; ^2^Department of Pathology, College of Veterinary Medicine, Suez Canal University, Ismailia, Egypt; ^3^Department of Clinical Studies, College of Veterinary Medicine, King Faisal University, Al-Ahsa, Saudi Arabia; ^4^Department of Biomedical Sciences, College of Veterinary Medicine, King Faisal University, Al-Ahsa, Saudi Arabia; ^5^Department of Pharmacology, Faculty of Veterinary Medicine, Kafrelshikh University, Kafrelshikh, Egypt; ^6^Department of Veterinary Pathobiology, School of Veterinary Medicine & Biomedical Sciences, Texas A&M University, College Station, TX, United States

**Keywords:** ameloblastoma, dromedary camel, odontogenic tumor, maxilla, odontogenic fibroma, squamous cell carcinoma

## Abstract

Four camels (*Camelus dromedarius*) presented to the Veterinary Teaching Hospital at King Faisal University with maxillary masses. On radiographs, the masses were multicystic and expanded the maxillary bone. The tumors were diagnosed by histopathologic examination as conventional ameloblastoma, two cases as intraosseous squamous cell carcinoma, and central odontogenic fibroma with ossification. To the authors’ knowledge, this is the first report of ameloblastoma in a camel, the first detailed description of maxillary squamous cell carcinoma in camels, and the first report of central odontogenic fibroma in any animal species.

## Introduction

Odontogenic neoplasms are uncommon in veterinary medicine (1). The classification of these tumors is complex and based on the presence of one or more odontogenic tissue types: odontogenic epithelium, mineralized dental matrices, and ectomesenchymal tissues of the dental papilla and dental follicle. Odontogenic neoplasms can be categorized into four main groups (2–4). The first group, tumors composed of odontogenic epithelium and fibrous stroma, includes conventional ameloblastoma, canine acanthomatous ameloblastoma, ameloblastic carcinoma, and amyloid-producing ameloblastoma. The second group, tumors composed of odontogenic epithelium and ectomesenchyme of the dental papilla and follicle, includes ameloblastic fibroma and feline inductive odontogenic tumor. The third group, odontogenic tumors composed of odontogenic epithelium, ectomesenchyme of the dental papilla and mineralized dental matrices, includes odontoma (complex and compound), ameloblastic fibro-odontoma (AFO), odontoameloblastoma, and cementoma. The fourth group, tumors derived from the tissues of the periodontal ligament, includes central and peripheral odontogenic fibroma. The occurrence of cells of multiple lineages within odontogenic tumors is thought to occur when neoplastic odontogenic epithelium induces adjacent cells to differentiate into ectomesenchymal tissues. Some odontogenic tumors, including canine acanthomatous ameloblastoma, amyloid-producing odontogenic tumor, and feline inductive odontogenic tumor, are unique to animals. On the other hand, some human odontogenic tumors, such as adenomatoid ameloblastoma and central odontogenic fibroma, are not recognized in veterinary medicine. Odontogenic fibroma is a rare odontogenic tumor characterized histologically by the presence of varying amounts of inactive-looking odontogenic epithelium embedded in a mature fibrous stroma. Osteoid, dentinoid, or cementum-like foci are usually present within the neoplasm, and it can be further classified into central (intraosseous) odontogenic fibroma and peripheral (extraosseous) odontogenic fibroma, depending on its location. In human medicine, peripheral odontogenic fibroma is more common than central odontogenic fibroma (5).

Neoplasia has been infrequently reported in Old World camelids. Documented neoplasms in the dromedary camel include nailbed squamous cell carcinoma, carpal chondrosarcoma, renal cell carcinoma, ovarian teratoma, lymphocytic leukemia, squamous cell carcinoma in the maxilla, and bronchoalveolar adenocarcinoma (6–12). In the Bactrian camel, reported tumors are limited to histiocytic sarcoma, meningioma, and gastric adenocarcinoma (13, 14). This report describes the clinical, radiographic, and pathologic features of four maxillary tumors in the dromedary camel. To the best of the authors’ knowledge, this is the first report of ameloblastoma in a camel, the first detailed description of maxillary squamous cell carcinoma in a camel, and the first report of central odontogenic fibroma in any animal species.

## Materials and methods

### Animals and sample collection

Four dromedary camels (*Camelus dromedarius*) presented to the Veterinary Teaching Hospital at King Faisal University with jaw masses. Blood samples were routinely collected from all animals. Radiographs of the head were taken, and representative tissue specimens were collected either by surgical excision or with biopsies taken using a Mickele trephine. Collected tissues were preserved in 10% neutral buffered formalin. Some specimens were decalcified in 15% formic acid as needed for sectioning. Tissues were processed routinely, embedded in paraffin wax, sectioned at 5 μm, and stained with hematoxylin and eosin (HE). Masson’s trichrome and Congo red stains were also done on selected cases.

### Immunohistochemistry

Immunohistochemical staining for pancytokeratin (clone AE1/AE3; 1:300 dilution, Biocare Medical, Pacheco, CA), and vimentin, (clone V9; 1:1000 dilution, Agilent Technologies, Santa Clara, CA), were performed on all cases. Camel skin was used as a positive control for the pancytokeratin, and goat liver and intestine were used as a positive control for the vimentin. 4 μm sections of the tumors were deparaffinized and treated with heat-induced epitope retrieval. Endogenous peroxidase was quenched with 3% hydrogen peroxide for 15 min, and sections were rinsed in 0.05 M trisbuffered saline solution (TBS/Tween 20). Nonspecific binding was blocked with normal goat serum (10% in TBS/Tween 20) for 15 min. Subsequently, all slides were incubated with primary antibody at the appropriate dilution and time and then rinsed. Next, sections were incubated with the specific secondary antibodies, rinsed 5 min in TBS/Tween 20, and color developed with 3-amino-9-ethylcarbazole reagent (ready-to-use, Dako North America) for 5–15 min, as appropriate. Sections were then rinsed in running tap water for 5 min and counterstained with Mayer’s hematoxylin (Sigma- Aldrich, St. Louis, MO) for 5 min.

## Results

### Animal demographics and relevant clinical history

The age, sex, breed, location of the lesion, and clinical histories of the four camels are shown in Table 1. All four animals were in fair body condition, and serum chemistry panels and complete blood counts (CBC) were within normal limits.

**Table 1:** Data and clinical history for the four camels.

Case	Age (years)	Sex	Breed	Location of the tumor	History
1	5	F	Wadha	R. maxilla	Four month history of growing mass that was interfering with mastication. The camel had hypersalivation and ulceration of the oral mucosa.
2	8	F	Majateer	R. maxilla	Five month history of facial swelling
3	6.5	F	Majateer	R. maxilla	Two month history of facial swelling, decreased chewing ability, and halitosis
4	9.5	F	Majateer	L. maxilla	Five month history of rapidly growing mass; regrew rapidly following surgical excision

### Pathologic and radiologic findings

**Figure 1:**
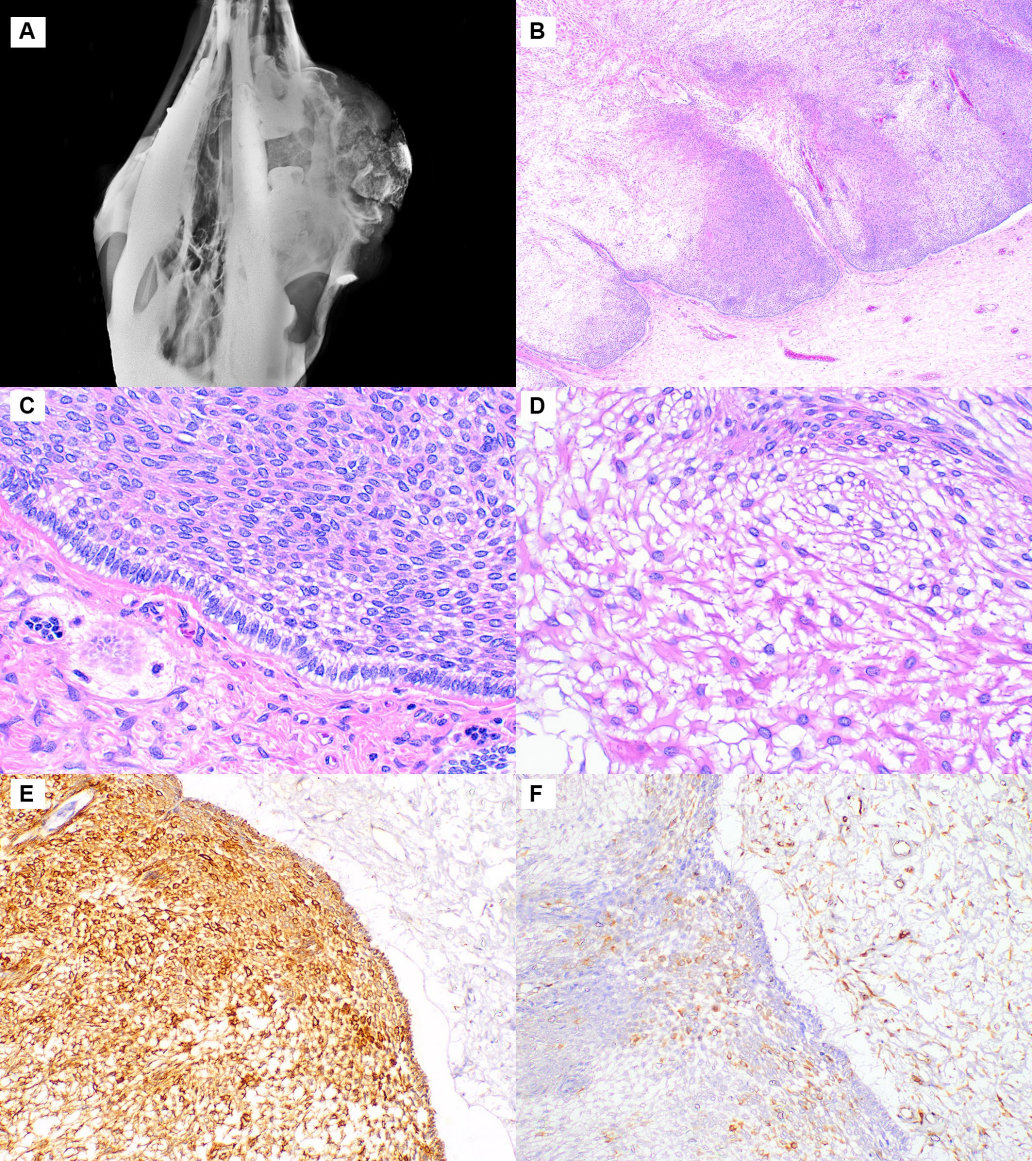
Ameloblastoma, maxilla, dromedary camel, Case 1. **(A)** Radiograph showing a large multicystic mass expanding the maxillary bone. **(B)** At low magnification, the neoplasm is composed of lobules lined by a layer of odontogenic epithelium, hematoxylin and eosin (HE). **(C)** Higher magnification of the odontogenic epithelium shows palisading of columnar cells with antibasilar nuclei and cytoplasmic vacuolation, HE. **(D)** Lobules are composed of stellate reticulum that is often keratinized, HE. **(E)** The odontogenic epithelium and stellate reticulum are strongly immunopositive for pancytokeratin, while the stromal cells on the right side are negative. Immunohistochemistry for pancytokeratin. **(F)** The stromal cells and a few of the stellate reticulum cells near the odontogenic epithelium are immunopositive for vimentin. Immunohistochemistry for vimentin.

In Case 1, the mass was in the right caudal maxilla. It measured 20 × 17 × 12 cm, was firm, and had an ulcerated surface. Radiographs revealed the mass to be multicystic and expanding the maxillary bone and sinus and replacing the third molar (Figure 1A). A 7 × 5 × 5 cm piece of the mass was surgically resected, and histopathologic examination revealed an infiltrative, unencapsulated neoplasm extending from the superficial to deep submucosa. The neoplasm was composed of large lobules and broad anastomosing trabeculae separated by abundant fibrovascular stroma (Figure 1B). The lobules and trabeculae were lined by 1–2 layers of palisading columnar to cuboidal cells with antibasilar nuclei and cytoplasmic vacuolation between the nuclei and basement membrane, consistent with odontogenic epithelium (Figure 1C). The center of the neoplastic lobules and trabeculae were variably cellular with less cellular areas composed of stellate cells with a small amount of cytoplasm and prominent intercellular bridges (stellate reticulum). In some areas, these cells had a moderate to large amount of brightly eosinophilic cytoplasm, consistent with keratinization (Figure 1D). In more densely cellular areas, the cells were spindle-shaped and arranged in short interweaving bundles and occasional whorls. Cellular atypia was lacking, and mitotic figures were not evident. Neoplastic lobules had frequent variably sized cysts that lacked a distinct lining and contained a small amount of globular proteinaceous material. The stroma was sparsely cellular with collagen bundles of varying thickness and multifocal accumulations of osteoid and woven bone. The overlying epithelium was moderately hyperplastic with multifocal erosion and infiltration of the superficial submucosa by moderate numbers of lymphocytes and plasma cells. The odontogenic epithelium and stellate reticulum were strongly immunopositive for pancytokeratin (Figure 1E) and mostly negative for vimentin, with the exception of some stellate reticulum cells immediately adjacent to the odontogenic epithelium (Figure 1F). The stromal cells between the epithelial lobules were immunopositive for vimentin and negative for pancytokeratin. The lesion was diagnosed as a conventional ameloblastoma.

**Figure 2:**
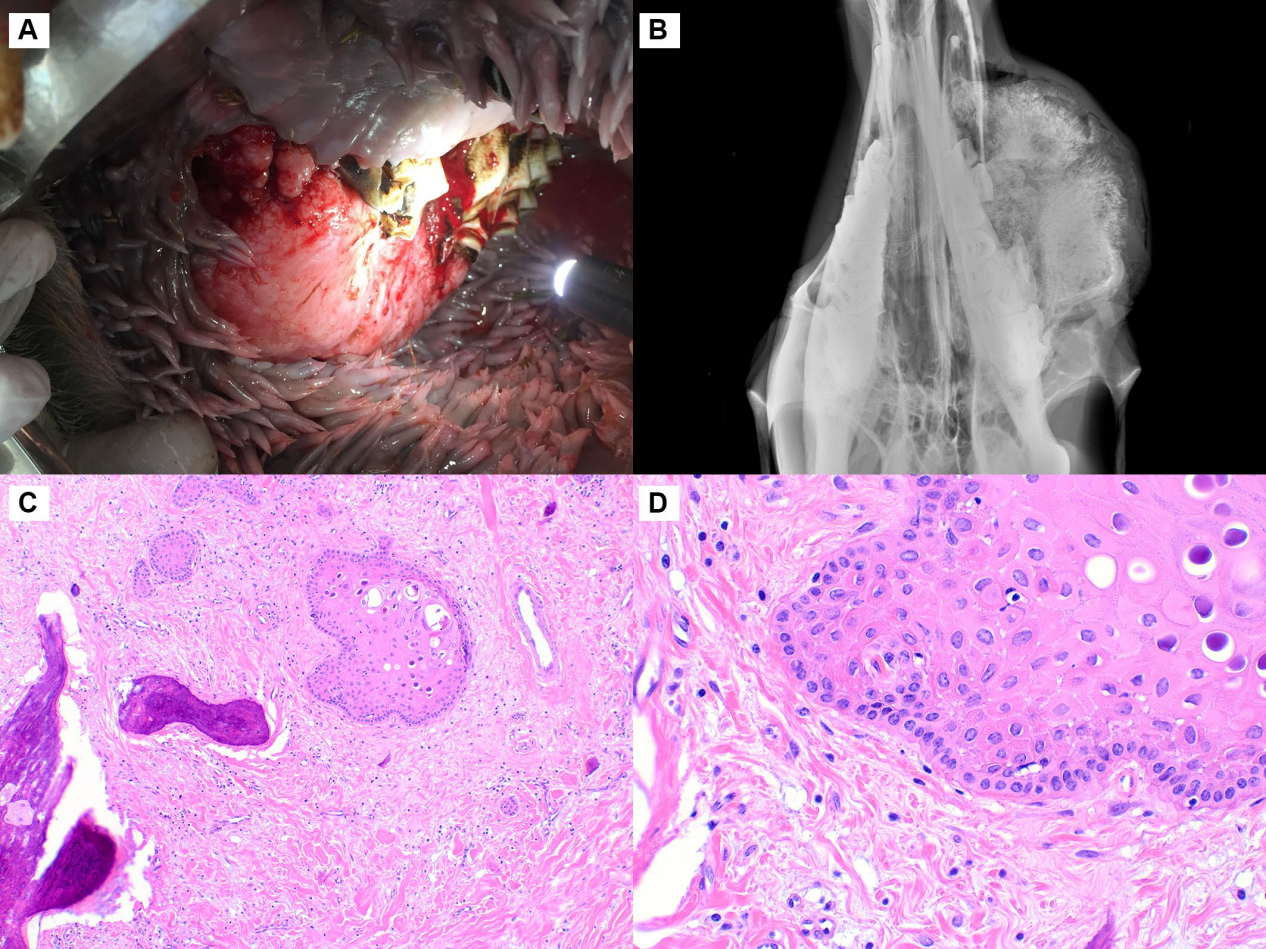
Central odontogenic fibroma, maxilla, dromedary camel, Case 2. **(A)** A firm, expansile mass is in the right caudal maxilla in the area of the second molar tooth. **(B)** Radiographically, the mass is uniformly radiopaque with multiple dark foci of osteolysis. **(C)** The neoplasm is sparely cellular, composed of abundant fibrous stroma with interspersed islands of odontogenic epithelium and lobules of woven bone, hematoxylin and eosin (HE). **(D)** At higher magnification, a sparse population of uniform spindle-shaped cells and abundant collagenous stroma is evident. The odontogenic epithelium exhibits peripheral palisading, HE.

In Case 2, the mass measured 16 × 8.5 × 5 cm and expanded the right caudal maxilla, surrounding the missing second molar tooth (Figure 2A). Radiographs showed a large, radiopaque, expansile mass extending from the right maxilla with multifocal osteolysis (Figure 2B). The neoplasm was sparsely cellular, composed predominantly of fibrous connective tissue containing monomorphic spindle-shaped cells lacking mitotic activity (Figures 2C,D). The cells had minimal cytoplasm and round to ovoid nuclei with finely stippled chromatin and inconspicuous nucleoli. In some areas, the connective tissue had a lightly basophilic, mucinous appearance. Randomly dispersed throughout the fibrous tissue, especially at the peripheral part of the mass in the sub-epithelial layer, were multifocal islands of odontogenic epithelium characterized by peripheral palisading with an antibasilar location of the nuclei (Figure 2D). Islands of well-differentiated woven bone occupied 70% of the tumor, particularly at its center. The epithelial islands were strongly immunopositive for pancytokeratin. With vimentin, the spindle-shaped cells stained strongly and there was mild staining near the periphery of the epithelial islands. Based on the presence of all three components (fibrous, osseous, and odontogenic epithelial), the diagnosis of central odontogenic fibroma with ossification was made.

**Figure 3:**
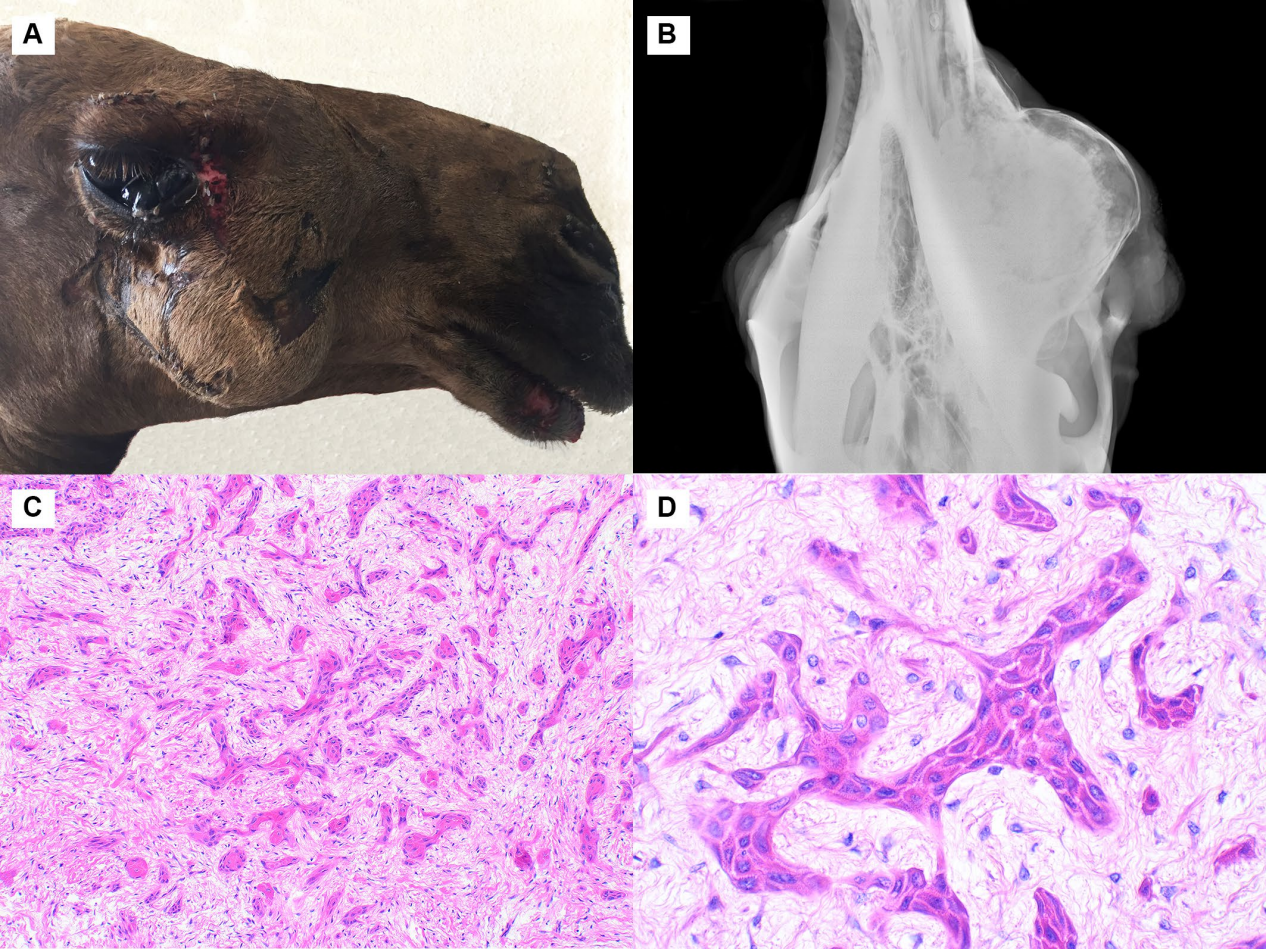
Squamous cell carcinoma, maxilla, dromedary camel, Cases 3 and 4. **(A)** A large firm mass is present in the right caudal maxilla. Case 3. **(B)** This radiograph shows an expansile radiopaque mass expanding the maxilla at the level of the third molar tooth. Case 3. **(C)** The neoplasm is composed of epithelial cells arranged in islands, cords, and plexiform ribbons within loose collagenous stroma. Case 3, hematoxylin and eosin (HE). **(D)** At high magnification, the cells are polygonal with brightly eosinophilic cytoplasm and prominent intercellular bridging. Case 4, HE.

Similar to Cases 1 and 2, the mass in Case 3 was in the right caudal maxilla. It was firm and measured 15x11x9 cm (Figure 3A). The skin overlying the lesion was multifocally ulcerated, and the third eyelid was protruded. Radiographs showed an expansile radiopaque mass expanding the maxilla at the level of the third molar tooth (Figure 3B). The mass consisted of epithelial cells arranged in islands, cords, and plexiform ribbons within a small to moderate amount of wispy collagenous stroma (Figure 3C). The pattern of the neoplastic cell islands was occasionally reminiscent of an ink drop pattern. Some islands exhibited central keratinization. The neoplastic cells were polygonal with prominent intercellular bridging, a small to moderate amount of eosinophilic and occasionally vacuolated cytoplasm, and centrally located nuclei with a finely stippled chromatin pattern. Neoplastic cells exhibited rare peripheral palisading. No atypia was observed, and the mitotic rate was less than 1 in 2.37 mm^2^. The epithelial cells were strongly immunopositive for pancytokeratin, and the stromal cells were strongly immunopositive for vimentin. The diagnosis of squamous cell carcinoma was made due to the lack of clear odontogenic features, including consistent peripheral palisading, basilar clearing, and the presence of a central stellate reticulum.

Case 4 involved a 19 × 10 × 7 cm rapidly-growing mass in the mid left maxilla. The mass regrew following surgical excision, and the animal was subsequently euthanized. Histologically, the tumor was infiltrative and composed of epithelial cells arranged in islands and cords within a moderate amount of loose collagenous stroma. A few larger islands and cords had central keratinization, and some had central cystic spaces. The cells were polygonal with a moderate amount of eosinophilic cytoplasm and exhibited prominent intercellular bridging (Figure 3D). The neoplastic cells occasionally displayed some cellular features reminiscent of odontogenic epithelium but lacked the classic peripheral palisading (in the majority of the sample), basilar clearing, and central stellate reticulum. Many small islands of woven bone were observed within the tumor. The immunohistochemistry staining pattern was identical to that described for Case 3. Like Case 3, the diagnosis was squamous cell carcinoma.

## Discussion

Ameloblastoma has been reported in many animal species, including dogs, cats, horses, sheep, cattle, New World camelids, rabbits, rodents, snakes, and salmon (1, 15–23). It is a locally invasive and destructive neoplasm that is associated with loss of teeth and bone, pathologic fractures, and facial deformities (22). Conventional ameloblastomas should exhibit all four of the cardinal features of odontogenic epithelium: palisading of the basilar epithelium, antibasilar nuclei within the basilar epithelial cells, clear zones within the cytoplasm of basilar epithelial cells, and polygonal cells with long intercellular bridges (resembling stellate reticulum) within the center of epithelial lobules (4). The tumor should lack the presence of the odontogenic mineralized matrices (dentin and enamel), the presence of which necessitates a diagnosis of ameloblastic odontoma or ameloblastic fibro-odontoma. The islands of mineralized material with embedded nuclei in this tumor were most suggestive of bone, but osteodentin with entrapped odontoblasts was another consideration. A diagnosis of ameloblastic fibroma was also considered, but the tumor lacked clear evidence of ectomesenchymal stromal induction.

Ameloblastoma has been previously reported in New World camelids (22–25). The age of the affected animals ranged from 9 months to 10 years, and the animals presented with a rapidly growing maxillary or mandibular mass. Three histologic variants have been described, including acanthomatous ameloblastoma in an alpaca, ameloblastic odontoma in a llama, and keratinized ameloblastoma in a llama. Similar to Case 1, those tumors had peripheral palisading of epithelial cells with anti-basilar nuclei and basilar vacuolation. Some areas in Case 1 had keratinization in the center of neoplastic trabeculae similar to that observed in the IIama with keratinized ameloblastoma.

Ameloblastoma is the second most common odontogenic neoplasm in humans (26). According to 2017 WHO Classification of Head and Neck Tumors, the neoplasm is classified as conventional ameloblastoma, unicystic ameloblastoma, and extraosseous/peripheral types (27). Conventional ameloblastoma has at least six histologic patterns: follicular, plexiform, acanthomatous, granular cell, basaloid, and desmoplastic. Ameloblastomas express many cytokeratins, including 13, 14, 19, and 56. They also express calretinin and CD56. The stromal component expresses vimentin, and vimentin expression can also be seen in the epithelial component. Peripheral cells show a higher expression of CK14, CK19, and CD56, while CK13 and calretinin are expressed in stellate reticulum-like cells. Ameloblastomas also express the early dental epithelial markers PITX2, MSX2, DLX2, RUNX1, and ISL1 (28, 29). The majority of human ameloblastomas have a mutation in MAPK/ERK pathway genes (27).

Ameloblastomas are benign. The malignant form, ameloblastic carcinoma, has been reported in the dog and horse (30–32). Despite their benign nature, ameloblastoma are usually locally aggressive and destructive tumors that interfere with food prehension and mastication. They also have a tendency to recur following incomplete surgical removal. In this case, the tumor regrew rapidly after surgical removal. To best of the authors’ knowledge, this is the first case of ameloblastoma reported in a dromedary camel. Although uncommon, it should be included in the differential diagnosis for maxillary and mandibular masses in this species.

Odontogenic fibroma is a benign tumor that originates from the mesodermal component of the tooth (dental follicle, periodontal ligament, or dental papilla) (33). It is characterized microscopically by the presence of dense to loose connective tissue intermingled with small clusters, nests, or cords of quiescent odontogenic epithelium resembling the dental lamina or the rests that are seen in normal dental follicles (34). Peripheral odontogenic fibroma (POF) is located in the gingiva adjacent to the teeth, and central odontogenic fibroma occupies the maxillary or mandibular bone (35). POF is common in dogs and is the only reported subtype in veterinary species (36). It is believed to arise from the periodontal ligament. POF also occurs rarely in cats and horses (37). Central odontogenic fibroma is a locally aggressive tumor that mostly occurs in the anterior maxilla and posterior mandible of middle-aged women. Less common tumor features include hard tissue formation, mineralized dentinoid or cementum-like calcification, deposition of amyloid-like protein, an associated central giant cell granuloma, and the presence of granular cells (38, 39). Case 2 appeared to arise out of the maxillary bone and included fibrous, osseous, and odontogenic epithelial components, therefore the diagnosis of central odontogenic fibroma with ossification was deemed appropriate. Central odontogenic fibroma has apparently not been previously reported in any animal species.

The histomorphologic features of case 3 and 4 are most consistent with oral squamous cell carcinoma (SCC). These two tumors lacked the classic features of odontogenic neoplasms, including prominent peripheral palisading of nuclei, basilar clearing, and central stellate reticulum. Some features, however, were considered unusual for SCC, including the expansile nature of the neoplasms on radiographs, the paucity of mitotic figures, the cystic appearance of some neoplastic trabeculae, and the presence of matrical islands resembling cementum. Additionally, the stroma surrounding the neoplastic cells in some areas looked similar to normal periodontal ligament, and the epithelial cells occasionally showed mild peripheral palisading reminiscent of odontogenic epithelium. Given these characteristics, an odontogenic origin for these two tumors cannot be completely ruled out. It is recognized that some odontogenic tumors can lose odontogenic features and closely resemble SCC (40, 41). The differential diagnosis for case 3 and case 4 included ameloblastic carcinoma, ameloblastic fibroma, and squamous odontogenic tumor. Unfortunately, these variants are poorly characterized in animals, and definitive immunohistochemical markers to differentiate these neoplasms are lacking (4).

Oral SCC is one of the most common human cancers, representing about 90% of all oral malignancies, but SCC arising from the gingiva is considered relatively rare, comprising less than 10% of oral cancers (42). Primary intraosseous squamous cell carcinoma originates in the mandibular or maxillary bone and has no initial connection to the oral mucosa. These tumors may arise from remnant odontogenic epithelium or from odontogenic cysts. Differentiation of these tumors from odontogenic tumors and SCC arising from the oral mucosa can be problematic (43). As in humans, SCC is one of the most frequently diagnosed malignant oral tumors in animals, especially dogs. In dogs, it is classified into tonsillar and nontonsillar types, and subtypes include conventional, basaloid, papillary, spindle cell, and adenosquamous carcinoma (44).

Further investigation is required to study the prevalence and prognosis of maxillary tumors in camels. In the future, greater refinement of morphologic criteria and development of more specific immunohistochemical markers will hopefully allow more accurate differentiation of this group of neoplasms.

## Data availability statement

The original contributions presented in the study are included in the article/supplementary material, further inquiries can be directed to the corresponding author.

## Ethics statement

The animal study was reviewed and approved by the Deanship of Scientific Research, King Faisal Univeristy. Written informed consent for participation was not obtained from the owners because specific consent procedures were not required for this study.

## Author contributions

AI, AA, FA-H, AN, MK, and MZ collecting samples and analysis of data. AI acquisition, analysis, and interpretation of data and drafted the manuscript. BP critically revised the manuscript. All authors contributed to the article and approved the submitted version.

## Funding

This work was supported by the Deanship of Scientific Research, Vice Presidency for Graduate Studies and Scientific Research, King Faisal University, Saudi Arabia [Grant No. 3315].

## Conflict of interest

The authors declare that the research was conducted in the absence of any commercial or financial relationships that could be construed as a potential conflict of interest.

## Publisher’s note

All claims expressed in this article are solely those of the authors and do not necessarily represent those of their affiliated organizations, or those of the publisher, the editors and the reviewers. Any product that may be evaluated in this article, or claim that may be made by its manufacturer, is not guaranteed or endorsed by the publisher.
